# Diuretic Activity of Compatible Triterpene Components of *Alismatis rhizoma*

**DOI:** 10.3390/molecules22091459

**Published:** 2017-09-06

**Authors:** Xue Zhang, Xiao-Yan Li, Na Lin, Wan-Li Zhao, Xiao-Qiang Huang, Ying Chen, Ming-Qing Huang, Wen Xu, Shui-Sheng Wu

**Affiliations:** 1 College of Pharmacy, Fujian University of Traditional Chinese Medicine, Fuzhou 350122, China; 18750717157@163.com (X.Z.); lixy8556@163.com (X.-Y.L.); linnayaoxue@163.com (N.L.); zhaowanlitcm@126.com (W.-L.Z.); 15880081619@163.com (X.-Q.H.); chenying578119815@163.com (Y.C.); hmq1115@126.com (M.-Q.H.); 2 Centre of Biomedical Research & Development, Fujian University of Traditional Chinese Medicine, Fuzhou 350122, China

**Keywords:** total triterpenes, *Alismatis rhizoma*, diuretic activity, component compatibility

## Abstract

*Alismatis rhizoma* (AR), the dried rhizoma of *Alisma orientale* Juzepzuk (Alismataceae), is a traditional Chinese medicine. AR is an important part of many prescriptions and is commonly used as a diuretic agent in Asia. This study aimed to evaluate the diuretic effects of total triterpene extract (TTE) and triterpene component compatibility (TCC, the mixture of alisol B 23-acetate, alisol B, alisol A 24-acetate, alisol A, and alisol C 23-acetate) of AR in saline-loaded rats. The optimal diuretic TCC of AR was optimized using a uniform design. Different doses (5, 20, and 40 mg/kg) of TTE and TCC groups (N1–N8) were orally administered to rats. Urinary excretion rate, pH, and electrolyte excretion were measured in the urine of saline-loaded rats. Results showed that TTE doses increased urine volume and electrolyte excretion compared with the control group. All uniformly designed groups of TCC also increased urine excretion. In addition, optimal diuretic TCC was calculated (alisol B 23-acetate: alisol B: alisol A 24-acetate: alisol A: alisol C 23-acetate 7.2:0.6:2.8:3.0:6.4) and further validated by saline-loaded rats. This study demonstrated that TTE presented a notable diuretic effect by increasing Na^+^, K^+^, and Cl^−^ displacements. The most suitable TTC compatible proportion of alisol B 23-acetate: alisol B: alisol A 24-acetate: alisol A: alisol C 23-acetate for diuretic activity was validated, and triterpenes were the material basis for the diuretic activity of AR.

## 1. Introduction

In recent years, an increase in the attempts to find natural sources of molecules with biological potential has been noticed. *Alismatis rhizome* (AR), the dried rhizome of *Alisma orientale* (Sam.) Juzepzuk (Alismataceae), is a natural medicine widely cultivated in China, Japan, Korea, India, and Europe. Traditionally, AR is known to exhibit diuretic, hyperlipidemic, inflammatory, antitumoral, and damp-heat clearing actions [1,2,3,4,5,6,7] and has been used for treating dysuria, edema, urinary tract infection, retention of fluid and phlegm, and vertigo for more than 1000 years [8]. As a folk medicine that has long been used to promote health and longevity, AR is now included in the Pharmacopoeia of China as a representative traditional Chinese herbal diuretic. AR is an important component of many famous Chinese formulae for diuretics in the Treatise on Febrile and Miscellaneous Disease (Shang Han Lun in Chinese) including Wu Ling San, Zhu Ling Tang, and Mu Li Ze Xie San [4], for usage as diuretics due to their low toxicity, high effectiveness, and minimal side effects. Natural medicine is a precious resource of therapeutically active compounds including terpenoids. Previous studies have reported the diuretic activity of different fractions (including petroleum ether, ethyl acetate, n-butanol, and remaining fractions) of the ethanol extract of AR. The results indicated that the ethyl acetate (100 and 400 mg/kg) fractions significantly increased urine output and electrolyte excretion. Furthermore, our previous study found fourteen terpenoids were isolated from the ethyl acetate fraction of AR, which indicated that terpenoids were the main ingredients of AR [9,10,11]. These triterpenoids (protostane-type) have attracted the most attention among the various constituents of AR, owing to their high content and various biological activities [4]. The ethanol extract of AR and alisol A 24-acetate exerts diuretic effects on animals [11], this ethanol extract contains mainly terpenoids, including alisol B 23-acetate, alisol B, alisol A 24-acetate, alisol A, and alisol C 23-acetate [12,13,14]. Alisol A, alisol B, alisol A 24-acetate, and alisol B 23-acetate were the first triterpenoids isolated from AR [15], before alisol C 23-acetate was also isolated from AR [16,17]. The most beneficial effects of AR are attributed to the presence of terpenoids [12,14,18]. Therefore, we speculated that these terpenoids may be the active compounds responsible for the observed diuretic effect. 

The curative effects of traditional Chinese medicine are not caused by single chemical entities, but result from their multi-ingredient prescription. The extent of compatibility depends on the integration of multitargets and multipathways, which are mediated by active component compatibility and exert corresponding terminal effects. Traditional Chinese medicine integration compatibility mainly manifests in component compatibility and specifically relates to multicomponent addition, competition and cooperation, and antagonistic action. Such treatment affects the performance of multicomponent multitargets and shows multicomponent sequence amplification and multicomponent selection of a dominant role.

**Figure 1:**
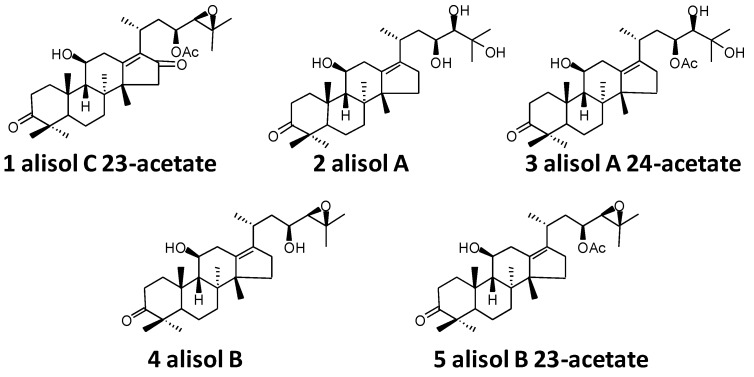
Chemical structures of five diuretic compounds used for compatibility studies.

Thus, the present study primarily aimed to evaluate the total triterpene extract (TTE) and triterpene component compatibility (TCC, including alisol B 23-acetate, alisol B, alisol A 24-acetate, alisol A, and alisol C 23-acetate, Figure 1) of the diuretic effect of AR in saline-loaded rats and to optimize the optimal diuretic TCC from AR.

## 2. Results

### 2.1. Determination of Triterpenes in Total Triterpene Extract (TTE)

**Figure 2:**
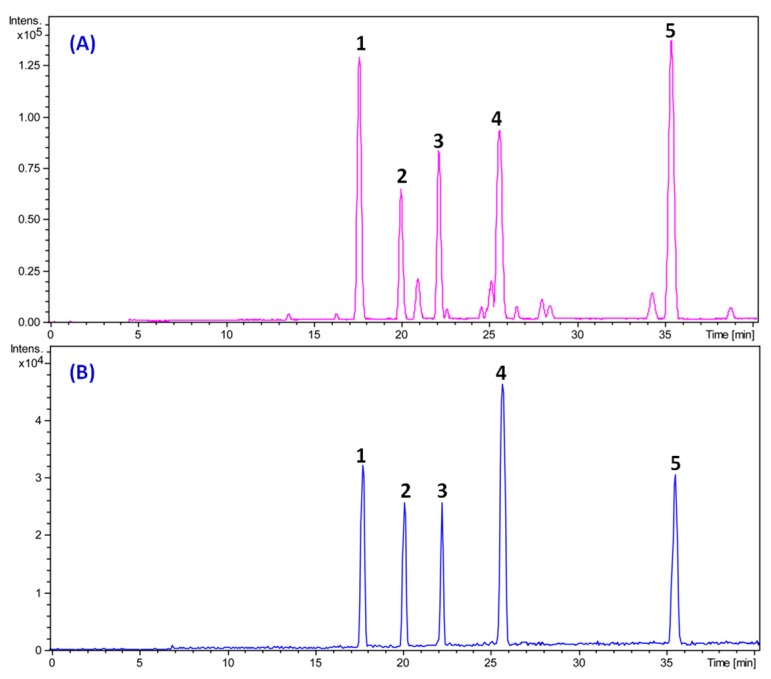
High-performance liquid chromatography-quadrupole time-of-flight mass spectrometry (HPLC-Q-TOF-MS) analysis of (**A**) total triterpene extract (TTE); and (**B**) reference substances: alisol C 23-acetate (1), alisol A (2), alisol A 24-acetate (3), alisol B (4), alisol B 23-acetate (5).

High-performance liquid chromatography-quadrupole time-of-flight mass spectrometry (HPLC-Q-TOF-MS) analysis showed that alisol A, alisol A 24-acetate, alisol B, alisol B 23-acetate, and alisol C 23-acetate were the major triterpenes in the TTE (Figure 2). Table 1 shows the triterpene composition of the TTE used in this study. The number of total triterpenoids containing five triterpenes in the TTE was 722.38 mg/g.

### 2.2. Urinary Volume and Electrolyte Excretion of the Diuretic Activity of TTE

**Figure 3:**
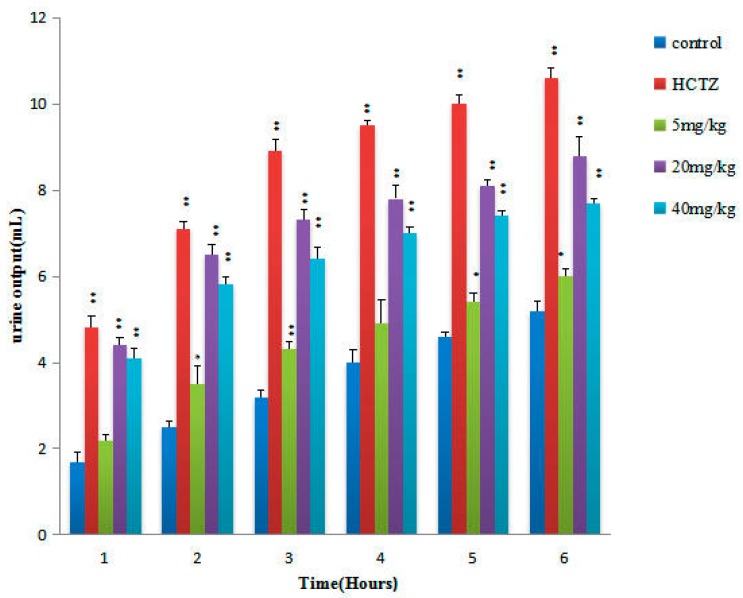
Time courses of diuretic activity activities of single oral doses (5, 20, and 40 mg/kg) of TTE, and the reference drug, HCTZ (20 mg/kg). The volume of excreted urine was measured at 1, 2, 3, 4, 5, and 6 h after administration. The cumulative values are reported as the mean ± SD for 10 rats in each group. *p < 0.05, **p < 0.01 compared with the controls.

The diuretic activity and electrolyte excretion results obtained after the oral TTE administration are shown in Figure 3 and Table 2, respectively. Different doses (5, 20, and 40 mg/kg) of TTE showed remarkable parallelism between urine output and electrolyte excretion of Na^+^ and Cl^−^. The 5 mg/kg of TTE increased Na^+^, Cl^−^, and K^+^ (by 17.8%, 15.1%, and 4.5%, respectively) when compared with the control group (Table 2). The 20 mg/kg doses of TTE also increased Na^+^, Cl^−^, and K^+^ (by 35.2%, 24.0%, and 20.1%, respectively) and achieved statistical significance compared with the control group. Furthermore, 40 mg/kg TTE significantly increased Na^+^, Cl^−^, and K^+^ (by 32.1%, 17.4%, and 12.1%, respectively). The urine pH results obtained after oral TTE administration are shown in Table 2. The pH values of the urine treated with 20 mg/kg doses of TTE were also significantly higher than those of the control group (*p* < 0.05). 

The results demonstrated that all the tested AR extracts increased urinary volume excretion. The TTE doses (5, 20, and 40 mg/kg) increased the urinary output in 6 h (15.4%, 69.23%, 48.08%, respectively), and the diuretic activity of high doses (40 mg/kg) were not superior to that of middle doses (20 mg/kg). The different doses may lead to different absorptions of main triterpene compositions, particularly TCC (different triterpene-to-triterpene ratios), which may exert different effects on diuretic activity. Therefore, the optimal diuretic TCC of AR was optimized using a uniformly designed experiment with five factors and eight levels, as shown in Table 3.

### 2.3. Urinary Volume and Electrolyte Excretion of the Diuretic Activity of Triterpene Component Compatibility (TCC)

**Figure 4:**
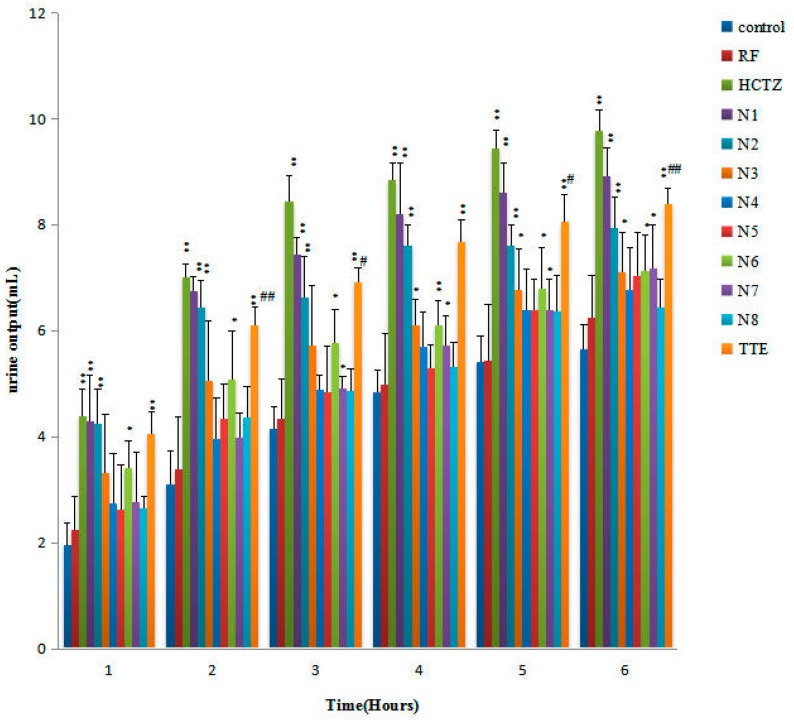
Time courses of diuretic activities of single oral doses (control, RF, HCTZ, N1, N2, N3, N4, N5, N6, N7, N8, and TTE) of TCC, and the reference drug HCTZ (20 mg/kg). The volume of excreted urine was measured at 1, 2, 3, 4, 5, and 6 h after administration. The cumulative values were reported as the mean ± SD for 8 rats in each group; * *p* < 0.05 when compared with the control group using ANOVA by Student´s *t*-test; ** *p* < 0.01 when compared with the control group using ANOVA by Student´s *t*-test; ^#^ *P* < 0.05 when compared with the N1 group using ANOVA by Student´s *t*-test; and ^##^ *p* < 0.01 when compared with the N1 group.

**Figure 5:**
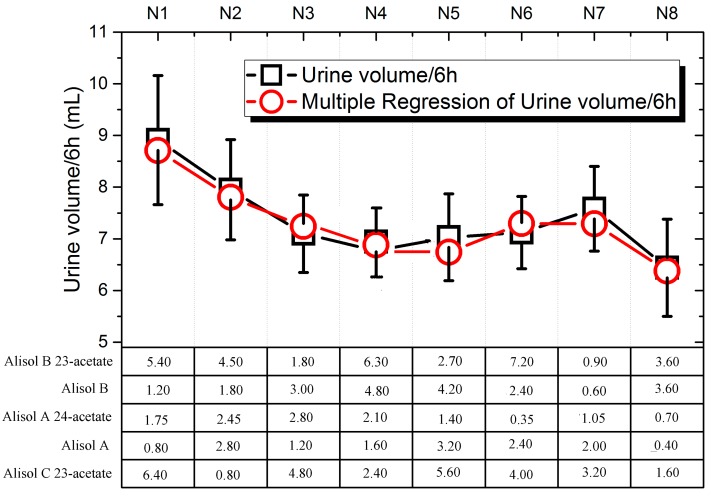
The effect of compatibility of alisol B 23-acetate, alisol B, alisol A 24-acetate, alisol A, and alisol C 23-acetate on diuretic activity based on uniform design. The multiple regression analysis of the compounds is included.

The results obtained after oral TCC administration are shown in Figure 4. The RF group (the remaining fraction without any compounds of alisol B 23-acetate: alisol B: alisol A 24-acetate: alisol A: alisol C 23-acetate after triterpene preparation from TEE) increased urinary excretion, but the increase was not statistically significant. Conversely, the hydrochlorothiazid (HCTZ) reached statistical significance in 6 h when compared with the control group. The N1–N8 groups also increased urine output by 57.70%, 40.71%, 25.67%, 19.82%, 24.42%, 26.02%, 27.08%, and 13.98%, respectively. The statistical method of selecting the optimum TTC of diuretic activity was performed using DPS 14.0 software. After comparing four statistical methods (Supplementary Table S1) recommended by the DPS 14.0 software, data were further analyzed by quadratic polynomial stepwise regression analysis to obtain the most suitable TTC regression equations (Figure 5): Y = 7.081 – 0.1689*X_2_ + 0.1403*X_5_ – 0.0316*X_1_*X_2_ + 0.1569*X_1_*X_3_ – 0.0998*X_2_*X_3_ + 0.0125*X_4_*X_5_ (X1: alisol B 23-acetate; X2: alisol B; X3; alisol A 24-acetate; X4: alisol A; X5: alisol C 23-acetate; and Y: urine output, diuretic activity). The correlation coefficient was R^2^ = 0.995. The most suitable ratio predicted for diuretic activity was as follows: alisol B 23-acetate: alisol B: alisol A 24-acetate: alisol A: alisol C 23-acetate at 7.2:0.6:2.8:3.0:6.4. The optimal combination was further verified three times as per standard laboratory practices, and the predicted optimal combination was close to the expected value of urine volume (Table 4). The optimal combination produced significant diuresis at 3–6 h, which was 65.1% of the total amount of urine.

After oral administration of TCC, urine electrolyte excretion presented considerable variation (Table 5) where N1 and N2 groups increased K^+^; N1–N5 and N7 groups increased Na^+^; and N1–N6 groups increased Cl^−^. The effects of HCTZ and RF for the AR group on electrolyte (Na^+^, K^+^, and Cl^−^) excretion in 6 h urine are summarized in Table 5. The optimal combination doses of TCC (Verify 1–3) enhanced the excretion of Na^+^ (*p* < 0.01), K^+^ (*p* < 0.05), and Cl^−^ (*p* < 0.05). Urine pH was considerably varied after the oral administration of TCC (Table 5). Furthermore, the pH values of urine treated with TCC were higher than those of the control group, except for N8. N1 and N2 also presented higher pH values (9.0% and 10.6%) compared with those of the control group, thereby showing a significant increase in pH values (*p* < 0.05 and *p* < 0.01). The optimal combination doses of TCC (Verify 1–3) also significantly increased urine pH.

## 3. Discussion

The diuretic effect of orally administered TTE and TCC of AR on normal Sprague-Dawley rats was evaluated where their pharmacological responses were compared with those produced by HCTZ, a common diuretic in clinical practices, and their effects on electrolyte balance determined. According to previous diuretic pharmacology studies of herbal medicines [19,20,21,22], saline-loaded normal rats have been generally used to evaluate the diuretic effects of herbal medicines. Establishing a stable diuretic animal model is very important in uniformly designed experiments of TCC research, thus, a saline-loaded normal rat model was chosen. Additionally, two parameters, namely increase in urine volume (water excretion) and net loss of solutes (i.e., electrolytes) in urine were also used [23].

The current study examined the diuretic effect of TTE doses and showed the different degrees of their diuretic effects. It was observed that electrolyte excretion (Na^+^, K^+^, and Cl^−^) was significantly increased by 20 and 40 mg/kg doses of TTE. Five mg/kg doses of TTE remarkably increased urine Na^+^ and Cl^−^ excretion. Furthermore, we observed that the diuretic activities of high (40 mg/kg) and low (5 mg/kg) doses were not superior to middle doses (20 mg/kg), which may demonstrate a non-linear relationship between the TTE dose and diuretic biological response. 

To clarify the actual material foundation of the diuretic effect of the TTE from AR, both HPLC-Q-TOF-MS and MS induced p-HPLC systems were used to determine and separate the major ingredients from the TTE. Consequently, five major triterpenoids (alisol B 23-acetate: alisol B: alisol A 24-acetate: alisol A: alisol C 23-acetate) were identified and separated. According to their contents, these TCC groups of AR were designed using a uniform design method. Each TCC group increased urinary volume excretion when compared with the control group, and the diuretic TCC of AR was optimized using uniformly design statistical analysis; however, the effect on K^+^ excretion was not significant. Additionally, it has been reported that the ethanol extract of AR and alisol A 24-acetate could increase Na^+^ excretion in rats and subsequently promote urine output [11]. Regarding the increase in urine output, the difference between the effects of each dose was indicated by the significant increase of Na^+^, Cl^−^, and K^+^ excretions when compared with those of the control group. Therefore, the diuretic action of the TTE of AR could be due to its interference with the Na^+^, K^+^, and Cl^−^ co-transport carrier in the luminal membrane of the thick ascending limb of the loop of Henle; which is similar to that of furosemide [24]. Thiazides initially enhance diuresis via inhibition of the kidney Na^+^–Cl^−^ cotransporter (NCC) [25]. It also appears to be related to the sodium–chloride co-transporter in the renal distal convoluting tubule, which is a site of sodium transport into the cortical interstitium [2]. The diuretic action TCC (only N1, N2) had a significant effect on K^+^ emissions, which was consistent with the conclusions of previous diuretic activity of the TTE.

The compatibility of the effective components of Chinese medicine is based on the theory of traditional Chinese medicine. With modern scientific guidance, traditional prescription compatibility theory, and basic, clear prescriptions of pharmacodynamic materials and mechanisms, their effects should be optimized to achieve the most desirable effects. Thus, the efficacy of traditional Chinese medicine results not from a single active ingredient, but from the compatibility of multiple components. 

In this study, we developed a TCC by combining five main active substances in the triterpenoids of AR. We found that the most suitable component ratio obtained in terms of diuretic activity was alisol B 23-acetate: alisol B: alisol A 24-acetate: alisol A: alisol C 23-acetate at 7.2:0.6:2.8:3.0:6.4. The urine volumes of rats from the RF group displayed no significant differences when compared with the control group. Accordingly, we concluded that the five main active ingredients (alisol B 23-acetate: alisol B: alisol A 24-acetate: alisol A: alisol C 23-acetate) of the TTE from AR exerted considerable diuretic effects. Moreover, modern pharmacology showed that 23-acetate alisol B and alisol A 24-acetate were the main ingredients contributing to the diuretic pharmacological activity of AR [4,11]. This finding was consistent with the results of the compatibility TCM study, which suggested that TCC may be the main active compound causing the diuretic activity of AR.

## 4. Materials and Methods 

### 4.1. Plant Material

AR was purchased from Fujian Jinshan Pharmaceutical Industrial Group Co., Ltd. (Fuzhou, China) and identified as *A. orientale* (Sam.) Juzepzuk by Prof. Shi-ming Fan (Fujian University of Traditional Chinese Medicine, Fuzhou, Fujian, China). A voucher specimen (No. CPH20151017) was deposited in the herbarium of the College of Pharmacy, Fujian University of Traditional Chinese Medicine, Fuzhou, Fujian.

### 4.2. Preparation of TTE and Quantification

According to the procedures previously described by our laboratory [26], the dried AR rhizomes were pulverized (24 mesh). The powder (1 kg) was extracted twice by decoction with 80% ethanol (20 L) for 1 h. The filtrate was concentrated to approximately 5 L under reduced pressure and then chromatographed on an AB-8 macroreticular resin (Nankai University Chemical Co., Tianjin, China) column using deionized water (20 L), 30% ethanol (10 L), 50% ethanol (10 L), and 75% ethanol (40 L) as eluents. To remove the tannins, the 75% ethanol fraction was concentrated to about 10 L under reduced pressure at 65 °C and chromatographed on a polyamide resin (60–80 mesh, SINOPEC, Hunan, China) column using deionized water (40 L) and 70% ethanol (60 L) as eluents. The 70% ethanol fraction was concentrated in vacuo (65 °C) and lyophilized using a freeze-drying system. The total acquired triterpene-enriched extract from AR was 12.8 g (yield 1.28%), which was subsequently dissolved with 2% Tween 80 to obtain the required dosage concentrations (5, 20, and 40 mg/kg).

To ensure the consistency of results with TTE, we analyzed the total triterpenes in the TTE using High-Performance liquid chromatography coupled with a quadrupole time-of-flight mass spectrometer (HPLC-Q-TOF-MS). In this system, a Bruker micrOTOF-Q II mass spectrometer (Bremen, Germany) was used with an Agilent 1260 Series LC System (Agilent Technologies, CA, USA), and a 1260-Quat quaternary solvent pump, 1260-Hip ALS auto-injector, and 1260-TCC column thermostat were included in the HPLC. The micrOTOF-Q II mass spectrometer was equipped with an electrospray ionization source and operated in positive mode. The optimized parameters were as follows: capillary, +3.5 kV; nebulizer pressure, 2.0 bar; drying gas N2 flow rate, 4.0 L/min; drying gas temperature, 180 °C; and ion transfer time, 80 μs. Additionally, argon was applied as the collision gas, and the collision energy was 30 eV to obtain the data of the fragment ions.

### 4.3. Preparation of TCC

Pure triterpenes from the TTE were separated using prepared high-pressure liquid chromatography (p-HPLC). The mass spectrometry (MS) induced p-HPLC system was performed on a Waters (Milford, MA) 2545 apparatus equipped with a 2767 fraction collector, SQD2 MS detector, and a Xbridge C18 (Waters, 5 µm, 150 mm × 19 mm) column. TTE was accurately weighed, dissolved in acetonitrile (500 mg/mL), and subjected to the p-HPLC system. The mobile phase consisted of purified water (A) and acetonitrile (B) with the following gradient elution program: 0–8 min, 35–65% B; 8–60 min, 35–25% A; and 60–70 min, 25–25% A; 20 mL/min. The detected ion sets were at 551 ([M + Na]^+^, alisol C 23-acetate), 513 ([M + Na]^+^, alisol A), 555 ([M + Na]^+^, alisol A 24-acetate), 495 ([M + Na]^+^, alisol B), and 537 ([M + Na]^+^, alisol B 23-acetate).Finally, 642 mg of alisol C 23-acetate powder (t_R_ 7.54 min), 324 mg of alisol A powder (t_R_ 12.30 min), 287 mg of alisol A 24-acetate powder (t_R_ 15.93 min), 482 mg of alisol B powder (t_R_ 19.18 min), and 725 mg of alisol B 23-acetate powder (t_R_ 25.22 min) were obtained, and their structures are shown in Figure 1. The remaining fractions (RF group, 1550 mg) of the TTE were also collected during the p-HPLC process. The RF group did not contain any of the separated triterpenes (alisol B 23-acetate, alisol B, alisol A 24-acetate, alisol A, and alisol C 23-acetate), which was confirmed by HPLC.

According to the uniformly designed method, all uniformly designed groups (Table 2) of TCC were prepared using pure triterpenes separated by the p-HPLC system by DPS 14.0 mathematical model statistical software (Zhejiang University, Zhejiang, China).

### 4.4. Reference Drug

Hydrochlorothiazide (HCTZ, Jiangsu Hailin Pharmaceutical Corporation, Jiangsu, China), a loop diuretic, was used as the reference drug (positive control). It was dissolved in 2% Tween 80 prior to administration.

### 4.5. Experimental Animals

Specific pathogen-free male Sprague-Dawley rats (180–220 g) were obtained from the Center of Laboratory Animal Science of Slack Laboratory Animal Co., Ltd. of Shanghai (SCXK (Shanghai) 2012-0002). All the rats were housed in the animal house of Fujian University of Traditional Chinese Medicine under standard environmental conditions (22 ± 2 °C, 40–60% relative humidity) with a 12 h light–dark cycle. The animals were also given free access to tap water and rat food. The experimental protocols for this study were approved by the Institutional Animal Care and Use Committee, and animals were kept according to the institutional ethical guidelines at Fujian University of Traditional Chinese Medicine.

### 4.6. Diuretic Activity of TTE

Diuretic activity was determined using the following reported method in reference [9]. The rats were deprived of food (but not water) for 12 h prior to testing. Their urinary bladders were emptied by gentle compression of the pelvic area and pulling their tails. Each rat was orally administered with 5 mL/100 g body weight (BW) of isotonic saline (NaCl, 0.9% w/v) to impose a uniform water load. After 30 min, the rats were randomly assigned into five groups (N = 10 per group) and treated orally in the following manner: Group 1: 1 mL/100 g BW of water; Groups 2–4: 5, 20, and 40 mg/kg BW of TTE, respectively; and Group 5: 20 mg/kg BW of HCTZ. Each rat was individually placed in a metabolic cage, and the cumulative urine output was determined at hourly intervals for 6 h. Electrolyte (Na^+^, K^+^, and Cl^−^) concentrations and pH were determined from 6 h urine samples of rats using a HC-9885 electrolyte analyzer (Shenzhen Aviation Medical Equipment Co., Ltd., Guangdong, China).

### 4.7. Diuretic Activity of TCC

Using the same rat model of the diuretic activity of TTE, the qualified rats were randomly assigned into 11 groups (N = 8 per group) and treated orally in the following manner: Group 1: 20 mL/kg BW of 2% Tween 80, Group 2: 20 mL/kg BW of HCTZ, Groups 3–10: uniformly designed group (N1–N8), and Group 11: 20 mL/kg BW of RF Group. TTE contained no 23-acetyl alisol B, alisol B, alisol A, 24-acetyl alisol A, and alisol 23-acetyl alisol C. The volume of collected urine was measured, and the ion concentration determined using the same approach as that used for the diuretic activity of the TTE.

### 4.8. Statistical Analysis 

Results were expressed as the mean ± S.E. (standard error of mean). Statistical differences between the negative control and test fractions were assessed using Analysis of Variance (ANOVA), and a Student’s *t*-test was subsequently conducted for multiple comparisons. All statistical analyses were performed using SPSS 16.0 with one-way ANOVA (SAS Institute Inc., Cary, NC, USA) and DPS 14.0 software (DPS Software, Herewood House, Enfield, UK). Compared with the control groups, *p*-values less than 0.05 were considered significant.

## Figures and Tables

**Table 1:** Characterization of five mainly triterpene constituents of the TTE by HPLC-Q-TOF-MS.

No.	t_R_	Molecular Ion	Error	Fragment Ions in Positive Mode	Molecular	Identity	Contents
	(min)	MS^1^	(ppm)	MS^2^	Formula		(mg/g)
1	17.53	529.3527 [M + H]^+^	0.8	511 [M + H-H_2_O]^+^, 469 [M + H-HAc]^+^, 451 [M + H-HAc-H_2_O]^+^, 415 [M + H-C_6_H_10_O_2_]^+^, 397 [M + H- C_6_H_10_O_2_-H_2_O]^+^	C_32_H_48_O_6_	alisol C 23-acetate	138.51
2	20.05	491.3139 [M + H]^+^	0.4	473 [M + H-H_2_O]^+^, 455 [M + H-2H_2_O]^+^, 437 [M + H-3H_2_O]^+^, 383 [M + H-H_2_O-C_4_H_10_O_2_]^+^	C_30_H_50_O_5_	alisol A	81.93
3	22.23	533.3833 [M + H]^+^	−0.6	515 [M + H-H_2_O]^+^, 497 [M + H-2H_2_O]^+^, 455 [M + H-HAc-H_2_O] ^+^, 437 [M + H-HAc-2H_2_O]^+^, 383 [M + H- C_6_H_12_O_3_-H_2_O]^+^	C_32_H_52_O_6_	alisol A 24-acetate	70.02
4	25.75	473.3631 [M + H]^+^	1.3	455 [M + H-H_2_O]^+^, 437 [M + H-2H_2_O]^+^, 383 [M + H-H_2_O-C_4_H_8_O]^+^	C_30_H_48_O_4_	alisol B	52.95
5	35.46	515.3732 [M + H]^+^	0.2	497 [M + H-H_2_O]^+^, 479 [M + H-2H_2_O]^+^, 437 [M + H-H_2_O-HAc]^+^, 383 [M + H-H_2_O- C_6_H_10_O_2_]^+^	C_32_H_50_O_5_	alisol B 23-acetate	153.67

t_R_ = retention time.

**Table 2:** Effects of oral administration of TTE and HCTZ; diuretic index, urinary electrolyte excretion, and pH value.

Group	Dose (mg/kg)	pH	Urine Electrolyte Concentration (mmoL∙L^−1^/6 h)			Saluretic Index			Na^+^/K^+^
			K^+^	Na^+^	Cl^−^	K^+^	Na^+^	Cl^−^	
control	−	6.85 ± 0.42	107.71 ± 20.40	110.77 ± 16.22	232.12 ± 20.15	1	1	1	1.03 ± 0.03
HCTZ	20	6.90 ± 0.72	120.11 ± 15.93	173.83 ± 22.90 *	299.54 ± 11.10 **	1.15	1.57	1.29	1.61 ± 0.08 **
TTE	5	6.97 ± 0.53	117.52 ± 16.23	130.43 ± 10.46 *	267.22 ± 11.09 *	1.09	1.18	1.15	1.11 ± 0.06 *
	20	7.28 ± 0.42 *	129.46 ± 18.13 *	149.75 ± 17.38 **	287.88 ± 29.55 **	1.20	1.35	1.24	1.16 ± 0.04 **
	40	7.06 ± 0.58	120.77 ± 8.11 *	146.28 ± 12.33 *	272.56 ± 17.11 **	1.12	1.32	1.17	1.21 ± 0.05 **

Note: Each value represents the mean ± SD; Diuretic index = volume extract group/volume control group; Saluretic index = extract group/control group; * *p* < 0.05, ** *p* < 0.01 compared with the controls.

**Table 3:** Different combinations of triterpene components of *Alismatis rhizoma*.

Factor	Alisol B 23-acetate /mg·kg^−^^1^	Alisol B/mg·kg^−^^1^	Alisol A 24-acetate /mg·kg^−^^1^	Alisol A/mg·kg^−^^1^	Alisol C 23-acetate /mg·kg^−^^1^
N1	5.40	1.20	1.75	0.80	6.40
N2	4.50	1.80	2.45	2.80	0.80
N3	1.80	3.00	2.80	1.20	4.80
N4	6.30	4.80	2.10	1.60	2.40
N5	2.70	4.20	1.40	3.20	5.60
N6	7.20	2.40	0.35	2.40	4.00
N7	0.90	0.60	1.05	2.00	3.20
N8	3.60	3.60	0.70	0.40	1.60

**Table 4:** The verified results of optimal TCC combination ratio.

			Urine Output/mL			
Group	1 h	2 h	3 h	4 h	5 h	6 h
Control	1.96 ± 1.01	3.11 ± 0.92	4.14 ± 0.93	4.84 ± 1.42	5.41 ± 0.80	5.65 ± 0.77
TTE	3.05 ± 0.59 *	6.51 ± 0.65 **	7.31 ± 0.62 **	7.87 ± 0.80 **	8.15 ± 0.91 **	8.59 ± 1.02 **
RF Group	2.24 ± 0.64	3.39 ± 0.99	4.35 ± 0.73	4.99 ± 0.95	5.43 ± 1.08	6.26 ± 0.78
HCTZ	4.40 ± 1.09 **	6.02 ± 1.65 **	7.04 ± 1.58 **	7.44 ± 1.83 **	8.54 ± 1.34 **	10.19 ± 1.07 **
Verify1	3.99 ± 0.67 **	5.85 ± 0.89 **	6.24 ± 0.91 **	7.40 ± 0.71 **	8.34 ± 1.08 **	9.49 ± 0.96 **
Verify2	3.73 ± 1.05 **	6.60 ± 1.01 **	7.63 ± 0.78 **	7.81 ± 0.88 **	8.61 ± 1.08 **	9.81 ± 0.89 **
Verify3	4.06 ± 0.69 **	5.71 ± 1.01 **	6.36 ± 0.78 **	7.87 ± 0.94 **	8.49 ± 0.77 **	9.63 ± 1.10 **

Each value represents the mean ± SD for 8 rats in each group; * *p* < 0.05 when compared with the control group using ANOVA by Student´s *t*-test; ** *p* < 0.01 when compared with the control group.

**Table 5:** Effects of oral administration of TCC, RF, and HCTZ on urinary volume excretion, electrolyte excretion, and pH value.

Group	Diuretic index	pH	Urine Electrolyte Concentration(mmoL∙L^−1^/6h)			Saluretic Index			Na^+^/ K^+^
			K^+^	Na^+^	Cl^−^	K^+^	Na^+^	Cl^−^	
Control	1	7.45 ± 0.35	102.02 ± 1.23	124.81 ± 8.23	234.64 ± 3.28	1	1	1	1.22 ± 0.21
TTE	1.52	8.09 ± 0.31 **	130.44 ± 16.9 **	195.18 ± 7.28 **	348.61 ± 8.64 **	1.27	1.56	1.48	1.49 ± 0.18
RF	1.11	7.49 ± 0.38	108.08 ± 9.90	164.94 ± 17.53	276.83 ± 9.52	1.06	1.32	1.18	1.52 ± 0.08 **
HCTZ	1.83	7.48 ± 0.66	112.79 ± 8.84	195.95 ± 7.86 **	358.31 ± 9.99 **	1.11	1.57	1.53	1.74 ± 0.12 **
N1	1.58	8.12 ± 0.25 *	131.01 ± 11.16 **	211.83 ± 16.00 **	363.70 ± 12.75 **	1.28	1.69	1.55	1.62 ± 0.16 **
N2	1.41	8.24 ± 0.18 **	141.45 ± 1.42**	184.76 ± 12.43 **	359.05 ± 13.87 **	1.39	1.48	1.1	1.30 ± 0.06
N3	1.26	7.80 ± 0.72	106.80 ± 31.11	166.92 ± 14.05 *	300.34 ± 11.37 **	1.05	1.33	1.28	1.56 ± 0.09 **
N4	1.20	7.54 ± 0.89	107.72 ± 13.76	152.05 ± 16.84 *	297.06 ± 9.26 **	1.06	1.21	1.26	1.41 ± 0.11 *
N5	1.24	7.48 ± 0.29	107.31 ± 18.53	161.88 ± 15.77 **	309.02 ± 24.57 *	1.05	1.29	1.32	1.51 ± 0.03 **
N6	1.28	7.50 ± 0.73	106.80 ± 11.12	140.12 ± 26.06	254.34 ± 11.22 **	1.05	1.12	1.08	1.31 ± 0.07
N7	1.29	7.54 ± 0.90	107.72 ± 19.77	152.17 ± 9.85 **	268.16 ± 28.45	1.06	1.22	1.14	1.41 ± 0.05 *
N8	1.16	7.18 ± 0.80	107.31 ± 10.54	131.88 ± 6.18	248.02 ± 18.98	1.05	1.06	1.06	1.23 ± 0.15
Verify1	1.56	8.29 ± 0.71 **	138.58 ± 11.16 **	201.06 ± 7.11 **	368.39 ± 9.07 **	1.36	1.62	1.57	1.45 ± 0.08 **
Verify2	1.63	8.17 ± 0.37 **	140.42 ± 1.64 **	199.56 ± 28.71 **	381.32 ± 9.78 **	1.38	1.59	1.62	1.42 ± 0.13 **
Verify3	1.65	8.42 ± 0.52 **	135.93 ± 6.62 **	210.56 ± 17.02 **	352.24 ± 6.34 **	1.33	1.68	1.50	1.56 ± 0.12 **

Each value represents the mean ± SD for 8 rats in each group; Diuretic index = volume extract group/volume control group; * *p* < 0.05 when compared with the control group using ANOVA by Student´s t-test; ** *p* < 0.01 when compared with the control group using ANOVA by Student´s t-test.

## Supplementary Materials

The following are available online.
