# Supplementary material for: Diuretic Activity of Compatible Triterpene Components of Alismatis rhizoma
Source: Molecules. 2017 Sep 6;22(9):1459. doi: 10.3390/molecules22091459 (PMC6151603; doi:10.3390/molecules22091459)
Supplement: Supplementary file 1 [file molecules-22-01459-s001.pdf]

Table S1 Analysis results of each method

factors	Quadratic polynomial stepwise regression	Stepwise regression item factors and interactions	Many factor and squared regression step by step	Stepwise regression
p	0.0112	0.0040	0.0364	0.2998
r	0.9999	0.9999	0.9998	0.9312
d	1.8234	1.7351	1.0412	1.8205

$p<0.05$ indicating a significant difference；

The closer the absolute value of r is closer to 1, the higher the degree of correlation between variables；

d statistics shows that residual is around 2 to obey normal distribution.
